# Salvianolic Acid B Suppresses Non-Small-Cell Lung Cancer Metastasis through PKM2-Independent Metabolic Reprogramming

**DOI:** 10.1155/2022/9302403

**Published:** 2022-04-23

**Authors:** Hong Zhang, Jianming Tang, Yu Cao, Tianhu Wang

**Affiliations:** Department of Thoracic Surgery, The Third Affiliated Hospital of Chongqing Medical University, Chongqing 401120, China

## Abstract

**Objective:**

Salvianolic acid B (Sal B) has been demonstrated to be a potential chemoprevention agent for several cancers. Herein, we investigated the pharmacological function of Sal B on non-small-cell lung cancer (NSCLC) metastasis.

**Methods:**

Two NSCLC cell lines (NCI-H2030 and NCI-H1650) were disposed of by 200 *μ*M Sal B or 10 *μ*M PKM2 agonist TEPP-46. Wound healing and transwell experiments were implemented for analyzing migratory and invasive capacities. Epithelial-to-mesenchymal transition (EMT) markers *β*-catenin and E-cadherin were measured via western blotting. Cellular bioenergetics were evaluated with glucose uptake, lactate production, enolase activity, cellular ATP levels, as well as seahorse-based oxygen consumption rate (OCR), extracellular acidification rate (ECAR) analysis. Metabolic reprogramming markers PKM2, LDHA, and GLUT1 were detected via western blotting and immunofluorescence.

**Results:**

The results showed that Sal B disposal weakened the migration and invasion of NCI-H2030 and NCI-H1650 cells and inactivated the EMT process according to downregulation of *β*-catenin and upregulation of E-cadherin. Sal B-treated NSCLC cells displayed decreased glucose uptake, lactate production, enolase activity, cellular ATP levels, OCR, and ECAR, indicating a reduction in metabolic reprogramming. Additionally, Sal B downregulated the expression of PKM2, LDHA, and GLUT1. TEPP-46 may reverse the inhibitory effect of Sal B on metastasis as well as metabolic reprogramming.

**Conclusion:**

Our findings provide evidence that Sal B enables to weaken NSCLC metastasis through PKM2-independent metabolic reprogramming, which sheds light on the promising therapeutic usage of Sal B in treating NSCLC.

## 1. Introduction

Non-small-cell lung cancer (NSCLC) is the most frequent and deadly type of primary lung malignancy, occupying 85% of all cases [1, 2]. It is histologically classified as adenocarcinoma, squamous cell carcinoma as well as large-cell carcinoma [3, 4]. Distant metastasis represents the dominating cause of mortality in NSCLC [5]. For resectable NSCLC tumors, primary treatment includes surgery with or without neoadjuvant therapy [6]. The mainstay of treatment for nonresectable tumors is radiotherapy and systemic treatments that are also offered for palliative treatment [7]. Systemic treatment contains conventional chemotherapy that is usually linked to remarkable side effects, targeted molecular treatment (such as tyrosine kinase inhibitors), as well as immunotherapies (such as immune-checkpoint inhibitors) [8]. Nevertheless, clinical drug resistance or nonresponse remains a challenge, which greatly hinders treatment success [9].

Many advances have been made in the comprehending of the pathophysiology of NSCLC metastasis. NSCLC is characterized by increased glucose and lactate utilization, and widespread heterogeneity in metabolic signaling [10]. Metabolic reprogramming represents a hallmark of cancer, and targeting metabolism has become a promising therapeutic strategy against NSCLC [11]. Pyruvate kinase may physiologically irreversibly catalyze phosphoenolpyruvate to pyruvate as a kinase in the final step of glycolysis [12]. Only tumor cells express the embryonic M2 isoform of pyruvate kinase (PKM2). As a crucial rate-limiting enzyme in glycolysis, PKM2 exerts an important function in the metabolic reprogramming of tumor cells [13]. The upregulation has been demonstrated in NSCLC, and secreted PKM2 facilitates NSCLC metastasis via activation of integrin *β*1/FAK signaling [14]. Thus, targeting PKM2 represents a potential therapeutic regimen of NSCLC.

Chemoprevention is considered a reasonable and attractive strategy to prevent or delay the development of NSCLC [15]. Chinese herbal medicine Salviae miltiorrhizae has been broadly applied in traditional Chinese medicine practice in treating cardiovascular and cerebrovascular diseases with minimal side effects [16]. Salvianolic acid B (Sal-B), extracted from the root of Salviae miltiorrhizae, is a major bioactive hydrophilic ingredient [17]. Several studies have demonstrated the anticancer pharmacological properties of Sal-B. For instance, Sal-B restrains glioma cells that are sensitive to radiotherapy through Fis-1-independent mitochondrial dysfunction [18]. Sal-B suppresses the growth of head and neck squamous cell carcinoma through cyclooxygenase-2 and apoptotic signaling [19]. Nevertheless, the pharmacological properties of Sal-B on anti-NSCLC remain indistinct. Here, we aimed to investigate the therapeutic effect of Sal-B on NSCLC metastasis. Our findings demonstrated that Sal B may weaken NSCLC metastasis with PKM2-independent metabolic reprogramming.

## 2. Materials and Methods

### 2.1. Cell Culture and Reagents

To investigate the therapeutic effect of Sal-B on NSCLC metastasis, two NSCLC cell lines (NCI-H2030 and NCI-H1650; ATCC, USA) were selected. All cells were grown in DMEM (Hyclone, USA) plus 10% fetal bovine serum (FBS; Hyclone) at 37°C in a humidified environment of 5% CO_2_. Sal B (purity ≥98%; Hongqiao Pharmaceutical Technology Research Institute Co., Ltd., Nanjing, China) was dissolved in absolute ethanol to 50 mM and stored at −80°C. Moreover, TEPP-46 was acquired from MedChemExpress Company (USA). NSCLC cell lines were treated with 10 *μ*M TEPP-46 for selectively activating pyruvate kinase M2 (PKM2).

### 2.2. Cell Counting Kit-8 (CCK-8)

NCI-H2030 and NCI-H1650 cells were inoculated into a 96-well plate lasting 24 hours. Thereafter, they were exposed to 0, 100, 200, 300, 400, 500, or 600 *μ*M Sal B lasting 24 or 48 hours. Following removing the cell culture medium with Sal B, the cells were treated with 100 *μ*L of cell culture medium plus 10% CCK-8 (Dojindo, Japan) lasting 4 hours. Optical densities were finally measured.

### 2.3. Wound Healing Assay

NCI-H2030 and NCI-H1650 cells were inoculated onto a 24-well plate. When subconfluence was reached, wound healing was made to the cell monolayers utilizing 200 *μ*L pipette tips. At 48 hours, wound healing was captured under light microscopy (×200; Leica, Germany), and the relative migration level was measured.

### 2.4. Transwell Assay

The Transwell assay with Matrigel (Sigma-Aldrich, USA) was implemented utilizing a 24-well plate with Transwell chambers (8 *μ*m pore; Sigma-Aldrich). The bottom chambers were added to with DMEM containing 20% FBS. 5 × 10^4^ NCI-H2030 and NCI-H1650 cells were inoculated into the upper chambers with serum-free medium. Migratory cells were fixed by 4% formaldehyde, stained with 1% crystal violet, followed by counting under light microscopy (×200; Leica, Germany).

### 2.5. Western Blotting

Protein extraction from NCI-H2030 and NCI-H1650 cells was implemented via the RIPA solution (P0013C; Beyotime, China). Protein concentrations were tested utilizing BCA assay kits (P0011; Beyotime). The extracted proteins were denatured in boiling water for 15-minutes. Thereafter, electrophoresis was carried out utilizing 10% SDS-PAGE gel for separation of protein molecules. Following protein transference onto PVDF membranes, the proteins were blocked in 5% nonfat milk at room temperature for 2 hours. Afterwards, incubation with primary antibody of *β*-catenin (1/1000; ab265591; Abcam, USA), E-cadherin (1/2000; ab231303), GAPDH (1/2500; ab9485), PKM2 (1/1000; #4053; Cell Signaling Technology, USA), phosphorylated PKM2 (p-PKM2; 1/1000; #3827; Cell Signaling Technology), lactate dehydrogenase A (LDHA; 1/1000; #3582; Cell Signaling Technology), and glucose transporter 1 (GLUT1; 1/1000; #73015; Cell Signaling Technology) was implemented at 4°C lasting 18 hours, followed by incubation with IgG-HRP goat anti-rabbit (1/3000; #7074; Cell Signaling Technology) for 2 hours. Signals were developed with ECL (Sigma-Aldrich) and normalized.

### 2.6. Glucose Uptake and Lactate Production

Glucose uptake was determined utilizing the glucose uptake assay kit (ab136955; Abcam) in accordance with the manufacturer's specification. Cell lysate was harvested as well as measured for lactate level utilizing the lactate assay kit (ab65330; Abcam) in accordance with the manufacturer's specification. The optical density value of cell lysate was measured after 24 hours. Lactate production was tested according to standard curves, followed by normalization to the cell lysate concentration.

### 2.7. Measurement of Enolase Activity

Enolase activity was measured with an enolase activity assay kit (ab241024; Abcam). In brief, cell lysate was mixed with the reaction buffer. After being incubated for 10 minutes at 25°C, the optical density value was quantified at 570 nm. Thereafter, each subsequent assay was carried out for 2 minutes until the optical density value of the most active specimens exceeded the highest standard. Enolase activity was finally calculated.

### 2.8. Enhanced ATP Assay

An enhanced ATP assay was carried out utilizing an enhanced ATP assay kit (S0027; Beyotime). The supernatant was added to 100 *μ*l of ATP detection solution. Thereafter, the RLU values were recorded through a Promega Glomax 20/20 luminometer. A standard curve was drawn according to the RLU values of ATP at concentrations of 0, 0.01, 0.05, 0.1, 0.5, 1, 5, and 10 nmol/L. The protein concentrations were utilized for standardizing the results and are shown as ATP/protein (nmol/mg).

### 2.9. Seahorse Analysis

Oxygen consumption rate (OCR) and extracellular acidification rate (ECAR) were detected utilizing XF24 extracellular analyzer (Seahorse Bioscience, USA). The 24-well cell culture microplate coated with Corning® Cell-Tak™ Cell as well as tissue adhesive was used for allowing adhesion of suspended cells. Thereafter, sequential compounds containing 1 *μ*M oligomycin, 0.5 *μ*M carbonyl-cyanide p-trifluoromethoxy phenylhydrazone (FCCP), and 0.5 *μ*M antimycin A (antiA)/rotenone (Rot), were added to the microplate for testing mitochondrial respiration. Furthermore, sequential compounds containing 10 mM glucose, 1 *μ*M oligomycin, and 50 mM 2-deoxyglucose (2-DG), were added for testing glycolytic activity.

### 2.10. Immunofluorescence

5 × 10^4^ NCI-H2030 and NCI-H1650 cells were inoculated into chamber slides. They were fixed with 4% paraformaldehyde (P0099; Beyotime) for 15 minutes, as well as permeabilized with 0.1% Triton X-100 (P0096; Beyotime) for 15 minutes. After blocking with 10% goat serum at room temperature, incubation with primary antibodies targeting PKM2 (1/100; #4053; Cell Signaling Technology) and GLUT1 (1/100; #73015; Cell Signaling Technology) was implemented at 4°C, as well as ALexa Fluor 488 conjugated secondary antibody (1/500; #4412; Cell Signaling Technology) and Hoechst 33342 labeling (1/500; #4082; Cell Signaling Technology). The results were imaged under confocal microscopy (×200; Leica).

### 2.11. Statistical Analysis

Statistical analysis was implemented via GraphPad Prism 8 software (San Diego, USA). The statistical comparison was carried out utilizing Student's *t*-test, one-way analysis of variance (ANOVA) or two-way ANOVA. The data are displayed as the mean ± standard deviation from at least three independent experiments. *P*values <0.05 were considered statistical significance.

## 3. Results

### 3.1. Sal B Suppresses Migration and Invasion of NSCLC Cells

**Figure 1:**
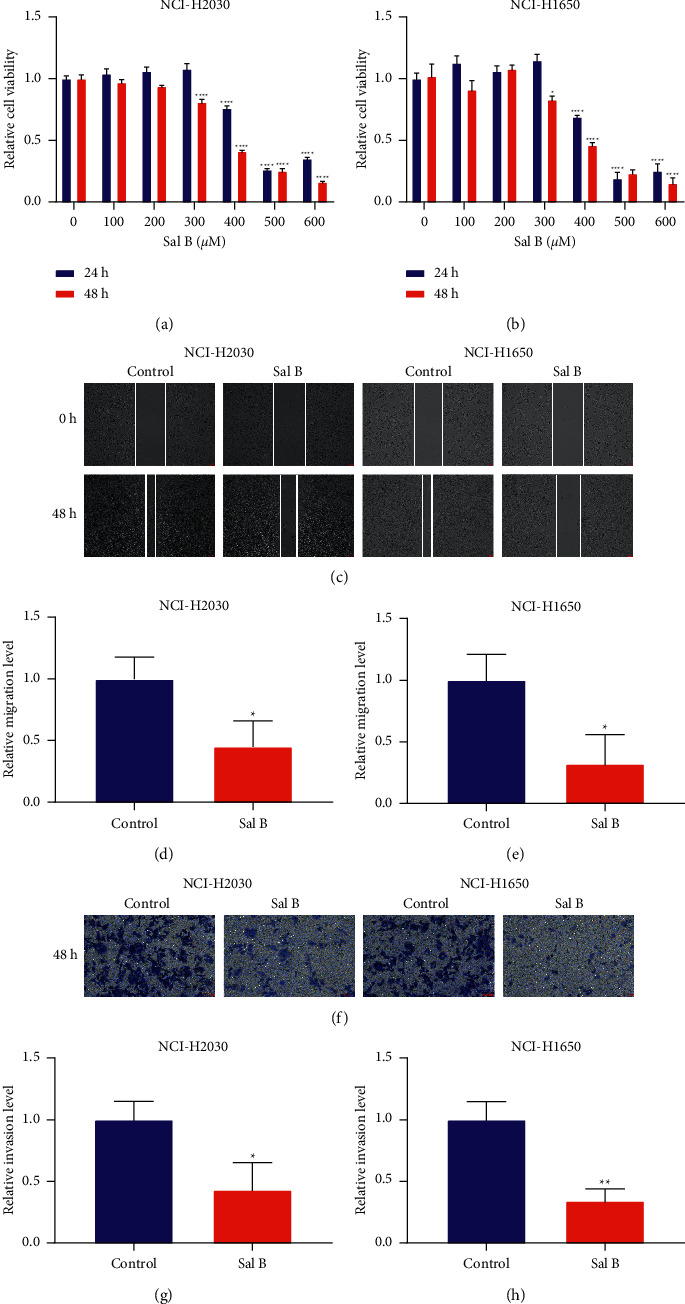
Sal B suppresses migration and invasion of NSCLC cells. (a, b) Cell viability of NCI-H2030 and NCI-H1650 cells that were exposed to a series of concentrations of Sal B lasting 24 or 48 hours through CCK-8. (c–e) Wound healing for assessing migration of 200 *μ*M Sal B-treated NCI-H2030 and NCI-H1650 cells. Bar = 50 *μ*M. (f–h) Evaluation of invasion of 200 *μ*M Sal B-treated NCI-H2030 and NCI-H1650 cells through transwell. Bar = 50 *μ*M. ^*∗*^*P* < 0.05; ^*∗∗*^*P* < 0.01; ^*∗∗∗∗*^*P* < 0.0001.

For investigation of the therapeutic function of Sal B, two NSCLC cells (NCI-H2030 and NCI-H1650) were exposed to distinct concentrations of Sal B for 24 or 48 hours. As shown in CCK-8, at 24 hours of exposure, when the concentration of Sal B reached 400 *μ*M, the cell viability of NCI-H2030 and NCI-H1650 cells displayed a significant reduction compared with controls (Figures 1(a) and 1(b)). At 48 hours of exposure, cell viability was remarkably decreased until the concentration reached 300 *μ*M which was higher than controls. This demonstrated that Sal B exposure enabled to weaken the proliferative capacity of NSCLC cells. We further evaluated the therapeutic function of 200 *μ*M Sal B on NSCLC metastasis. The data from the wound healing assay showed that the migration capacity of NCI-H2030 and NCI-H1650 cells was remarkably weakened by 200 *μ*M Sal B (Figures 1(c)–1(e)). Additionally, 200 *μ*M Sal B significantly reduced the invasion capacity of NCI-H2030 and NCI-H1650 cells (Figures 1(f)–1(h)). Hence, Sal B treatment enabled to suppress NSCLC metastasis.

### 3.2. Sal B Weakens Epithelial-Mesenchymal Transition (EMT) Process in NSCLC Cells

**Figure 2:**
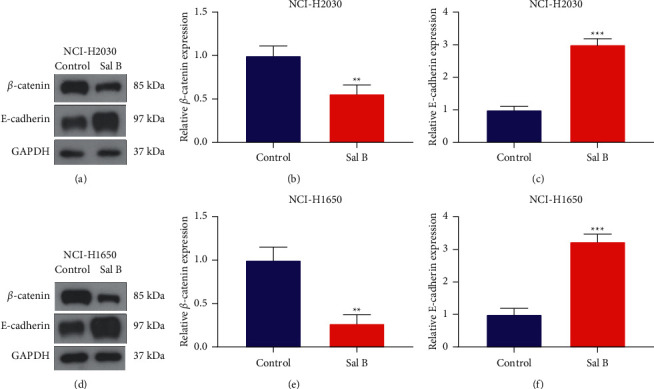
Sal B weakens EMT process of NSCLC cells. (a–c) Western blotting of the expression of *β*-catenin and E-cadherin in 200 *μ*M Sal B-treated NCI-H2030 cells. (d–f) Western blotting of the expression of *β*-catenin and E-cadherin in 200 *μ*M Sal B-treated NCI-H1650 cells. ^*∗∗*^*P* < 0.01; ^*∗∗∗∗*^*P* < 0.0001.

EMT, a reversible developmental genetic program for transdifferentiating polarized epithelial cells to mesenchymal cells, enables to trigger metastasis during the progression of NSCLC [20]. Further analysis showed that 200 *μ*M Sal B markedly reduced *β*-catenin expression as well as elevated E-cadherin expression in NCI-H2030 as well as NCI-H1650 cell lines (Figures 2(a)–2(f)), indicating that Sal B enabled to inactivate EMT process of NSCLC cells.

### 3.3. Sal B Weakens Metabolic Reprogramming of NSCLC Cells

**Figure 3:**
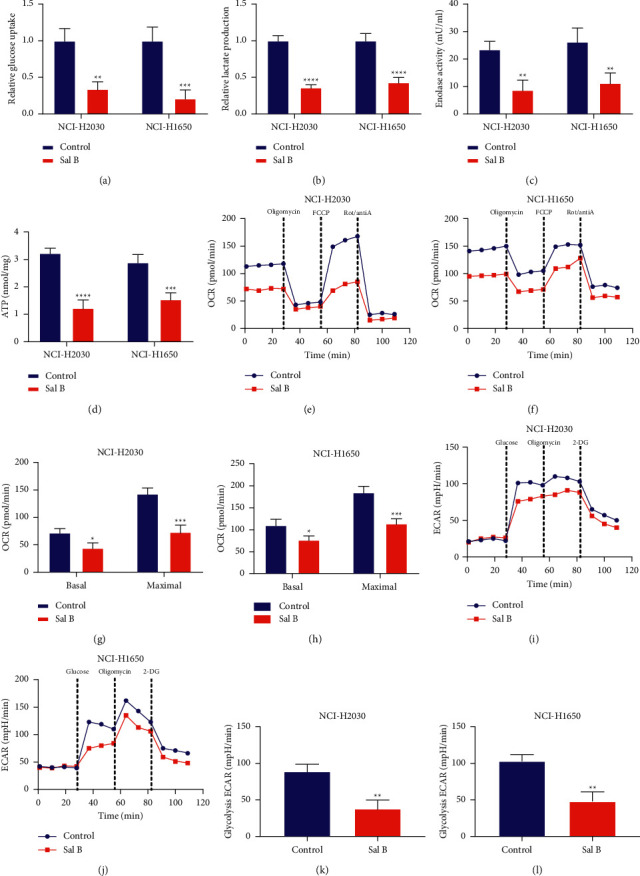
Sal B weakens metabolic reprogramming of NSCLC cells. (a–d) Glucose uptake, lactate production, enolase activity, as well as cellular ATP production of 200 *μ*M Sal B-treated NCI-H2030 and NCI-H1650 cells. (e, f) Measurement of OCR of 200 *μ*M Sal B-treated two NSCLC cells when exposure to oligomycin, FCCP, and Rot/antiA. (g, h) Quantification of basal and maximal OCR of 200 *μ*M Sal B-treated two NSCLC cells. (i, j) Measurement of ECAR of 200 *μ*M Sal B-treated two NSCLC cells after exposing to glucose, oligomycin, and 2-DG. (k, l) Quantification of glycolysis ECAR of above two NSCLC cells. ^*∗*^*P* < 0.05; ^*∗∗*^*P* < 0.01; ^*∗∗∗*^*P* < 0.001; ^*∗∗∗∗*^*P* < 0.0001.

Metabolic reprogramming has been widely recognized as a cancer hallmark [13]. Further analysis was implemented for evaluating the therapeutic function of Sal B on the metabolic reprogramming of NSCLC cells. Compared with controls, 200 *μ*M Sal B-treated NCI-H2030 and NCI-H1650 cells presented remarkably reduced glucose uptake, lactate production, enolase activity as well as cellular ATP production (Figures 3(a)–3(d)). To evaluate mitochondrial respiration, we measured OCR after adding sequential compounds containing 1 *μ*M oligomycin, 0.5 *μ*M FCCP, and 0.5 *μ*M Rot/antiA. As a result, 200 *μ*M Sal B prominently decreased OCR both in NCI-H2030 and NCI-H1650 cells than controls (Figures 3(e)–3(h)). We also evaluated glycolytic activity through measuring ECAR following sequential compounds containing 10 mM glucose, 1 *μ*M oligomycin, and 50 mM 2-DG. The data showed that 200 *μ*M Sal B-treated NCI-H2030 and NCI-H1650 cells displayed significantly decreased ECAR than controls (Figures 3(i)–3(l)). Altogether, Sal B enabled to weaken the metabolic reprogramming of NSCLC cells.

### 3.4. Sal B Inhibits PKM2-Mediated Metabolic Reprogramming of NSCLC Cells

**Figure 4:**
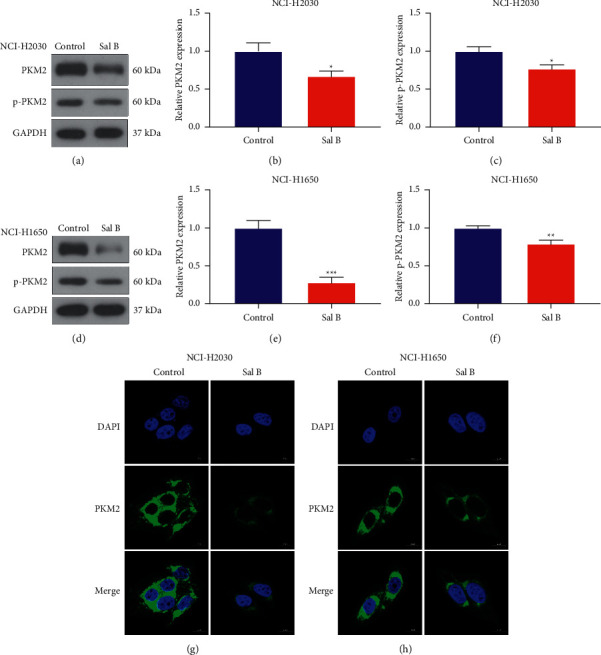
Sal B inhibits PKM2-mediated metabolic reprogramming of NSCLC cells. (a–c) Western blots for PKM2 as well as p-PKM2 expressions in 200 *μ*M Sal B-treated NCI-H2030 cells. (d–f) Western blots for PKM2 as well as p-PKM2 expressions in 200 *μ*M Sal B-exposed NCI-H1650 cells. (g, h) Immunofluorescence for investigating PKM2 expression in 200 *μ*M Sal B-exposed NCI-H2030 and NCI-H1650 cells. Bar = 10 *μ*M. ^*∗*^*P* < 0.05; ^*∗∗*^*P* < 0.01; ^*∗∗∗*^*P* < 0.001.

PKM2 is a central regulator of aerobic glycolysis of cancer cells, which accelerates lactate production as well as metabolic reprogramming [21]. Our western blotting showed that 200 *μ*M Sal B-treated NCI-H2030 and NCI-H1650 cells displayed remarkably reduced expression of PKM2 and p-PKM2 in comparison to controls (Figures 4(a)–4(f)). Immunohistochemistry also demonstrated that Sal B was able to decrease PKM2 expression in NCI-H2030 and NCI-H1650 cells (Figures 4(g) and 4(h)). Thus, Sal B inhibited PKM2-mediated metabolic reprogramming of NSCLC cells.

### 3.5. Sal B Restrains Metabolic Reprogramming-Relevant Genes LDHA and GLUT1 in NSCLC Cells

**Figure 5:**
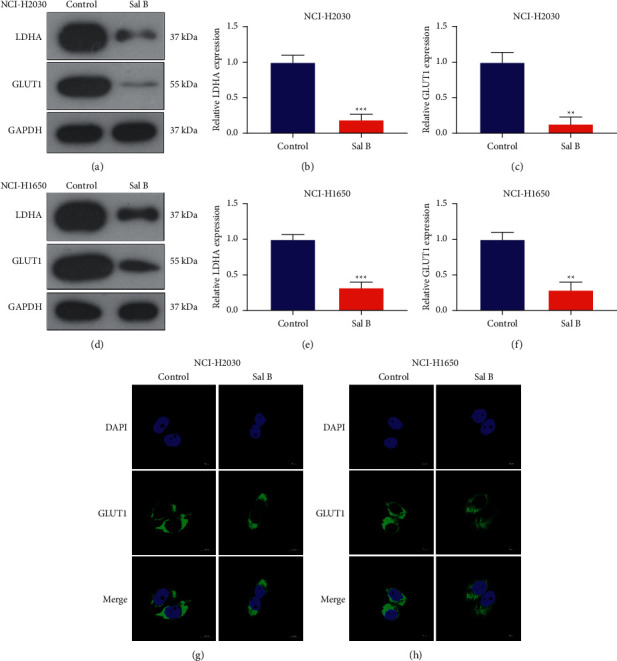
Sal B restrains metabolic reprogramming-relevant genes LDHA and GLUT1 in NSCLC cells. (a–f) Western blots for measuring LDHA and GLUT1 expressions in 200 *μ*M Sal B-treated NCI-H2030 as well as NCI-H2030 cells. (g, h) Immunofluorescence for investigating the expression of LDHA and GLUT1 in 200 *μ*M Sal B-exposed NCI-H2030 and NCI-H1650 cells. Bar = 10 *μ*M. ^*∗∗*^*P* < 0.01; ^*∗∗∗*^*P* < 0.001.

We also assessed the effects of Sal B on the metabolic reprogramming-relevant genes LDHA and GLUT1 in NSCLC cells. As a result, in comparison to controls, 200 *μ*M Sal B remarkably reduced the expression of LDHA and GLUT1 both in NCI-H2030 and NCI-H2030 cells (Figures 5(a)–5(f)). Immunohistochemistry also confirmed the decreased expression of GLUT1 in 200 *μ*M Sal B-treated NCI-H2030 as well as NCI-H2030 cells (Figures 5(g) and 5(h)).

### 3.6. Sal B Suppresses Migration and Invasion of NSCLC Cells via PKM2-Independent Metabolic Reprogramming

**Figure 6:**
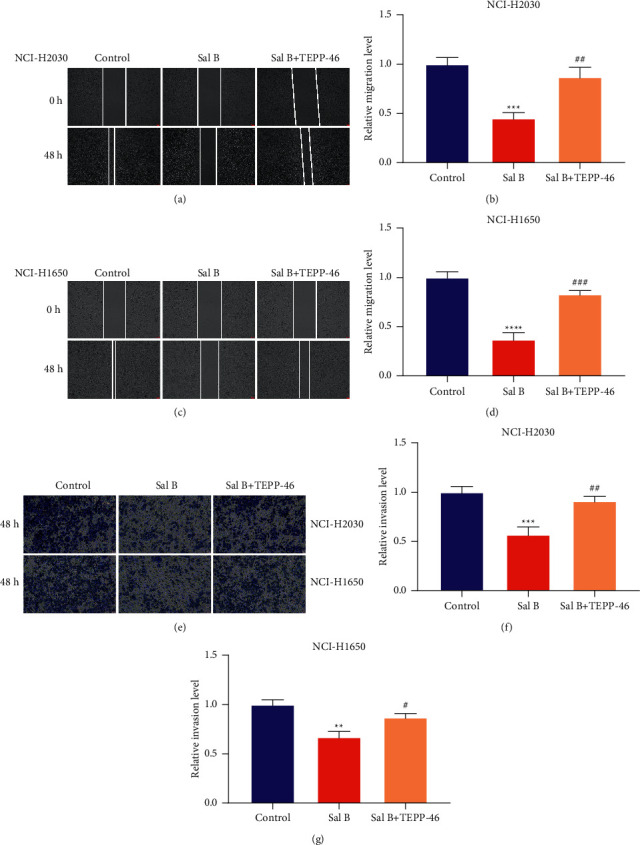
Sal B suppresses migration and invasion of NSCLC cells via PKM2-independent metabolic reprogramming. (a–d) Wound healing of the migration of NCI-H2030 and NCI-H1650 cells when exposure to 200 *μ*M Sal B or 10 *μ*M TEPP-46. Bar = 50 *μ*M. (e–g) Transwell for evaluating the invasion of NCI-H2030 and NCI-H1650 cells following exposure to 200 *μ*M Sal B or 10 *μ*M TEPP-46. Bar = 50 *μ*M. Compared with control group, ^*∗∗*^*P* < 0.01; ^*∗∗∗*^*P* < 0.001; ^*∗∗∗∗*^*P* < 0.0001. Compared with Sal B group, ^#^*P* < 0.05; ^##^*P* < 0.01; ^###^*P* < 0.001.

We conducted further analysis to demonstrate whether Sal B restrained NSCLC metastasis with PKM2-independent metabolic reprogramming. 10 *μ*M TEPP-46 was utilized for selectively activating PKM2 expression. As shown in wound healing, TEPP-46 markedly weakened the inhibitory effects of Sal B on the migration capacity of NCI-H2030 and NCI-H1650 cells (Figures 6(a)–6(d)). Additionally, transwell results demonstrated that TEPP-46 enabled to reverse the inhibitory effects of Sal B on the invasion capacity of NCI-H2030 and NCI-H1650 cells (Figures 6(e)–6(g)). Altogether, Sal B may suppress NSCLC metastasis via PKM2-independent metabolic reprogramming.

### 3.7. Sal B Weakens EMT Process of NSCLC Cells through PKM2-Independent Metabolic Reprogramming

**Figure 7:**
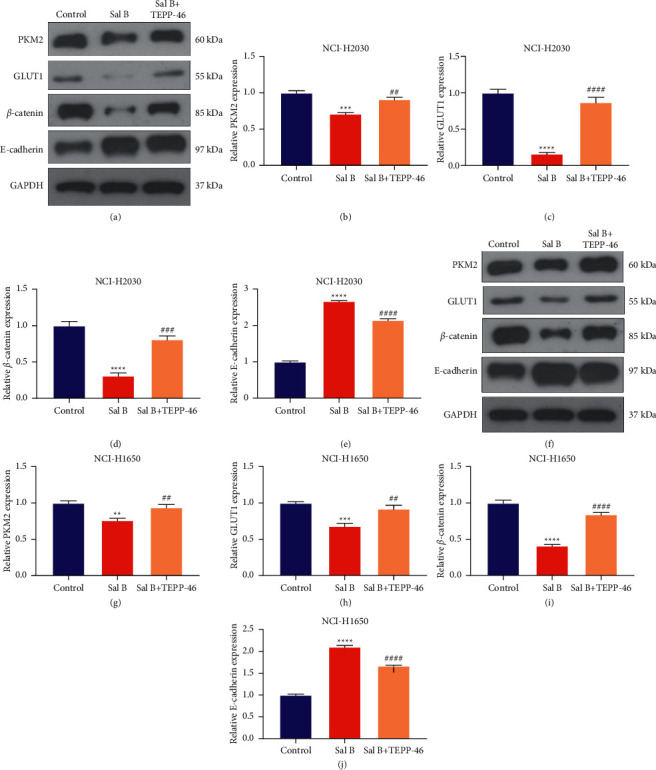
Sal B weakens EMT process of NSCLC cells through PKM2-independent metabolic reprogramming. (a–e) Western blotting for evaluating PKM2, GLUT1, *β*-catenin, and E-cadherin expressions in NCI-H2030 cells when exposure to 200 *μ*M Sal B or 10 *μ*M TEPP-46. (f–j) Western blotting of PKM2, GLUT1, *β*-catenin, and E-cadherin expressions in NCI-H1650 cells following exposure to 200 *μ*M Sal B or 10 *μ*M TEPP-46. Compared with control group, ^*∗∗*^*P* < 0.01; ^*∗∗∗*^*P* < 0.001; ^*∗∗∗∗*^*P* < 0.0001. Compared with Sal B group, ^##^*P* < 0.01; ^###^*P* < 0.001; ^####^*P* < 0.0001.

As expected, TEPP-46 was capable of enhancing PKM2 and GLUT1 expression in Sal B-exposed NCI-H2030 as well as NCI-H1650 cells (Figures 7(a)–7(j)), demonstrating that TEPP-46 markedly reversed the inhibitory effects of Sal B on metabolic reprogramming of NSCLC cells. Further analysis demonstrated that TEPP-46 administration enhanced *β*-catenin expression as well as decreased E-cadherin expression in Sal B-treated NCI-H2030 and NCI-H1650 cells (Figures 7(a)–7(j)). Thus, Sal B weakened EMT activation of NSCLC cells with PKM2-independent metabolic reprogramming.

## 4. Discussion

NSCLC is a fatal malignancy with a hallmark of abnormal metabolism [22]. Sal B represents a dominating water-soluble component extracted from Salvia miltiorrhiza [23]. Our data demonstrated that Sal B was capable of weakening NSCLC metastasis with PKM2-independent metabolic reprogramming.

Metastasis represents an extremely complex multistage process [24]. Tumor cells are detached from the extracellular matrix and then colonize surrounding environment, demonstrating that benign nodules transform to aggressive malignancies [25–27]. Sal B-treated NCI-H2030 and NCI-H1650 cells displayed reduced migration and invasion capacities, indicating the inhibitory effect of Sal B on NSCLC metastasis. Previous research has demonstrated that Sal B may restrain the migration and invasion of hepatocellular carcinoma through RECK/STAT3 signaling [28]. For crossing the basement membrane along with the basal layer, tumor cells are functionally and morphologically altered through activating EMT that has the features of upregulation of *β*-cadherin and downregulation of E-cadherin [29]. Sal B administration enabled the decrease *β*-cadherin expression as well as the enhancement of E-cadherin expression of NCI-H2030 and NCI-H1650 cells, demonstrating the inactivation of EMT in NSCLC cells. Previous evidence has proposed that Sal B is capable of suppressing EMT to alleviate drug resistance via AKT/mTOR signaling in gastric cancer [30].

Tumor cells usually reprogram the metabolism to effectively support cell proliferation and survival [31]. Herein, Sal B treatment markedly reduced glucose uptake, lactate production, enolase activity, cellular ATP production, OCR, and ECAR of NCI-H2030 and NCI-H1650 cells, demonstrating that Sal B weakened the metabolic reprogramming of NSCLC cells. Limited evidence has proposed the suppression effect of Sal B on metabolic reprogramming. For instance, Sal B may weaken glycolysis in oral squamous cell carcinoma through PI3K/AKT/HIF-1*α* signaling [15]. Additionally, Sal B reduces M1-polarized macrophages in myocardial ischemia/reperfusion damage through weakening mTORC1-dependent glycolysis [32–34]. PKM2 is a crucial regulator of metabolic reprogramming, which may catalyze the synthesis of pyruvate from phosphoenolpyruvate and facilitate glycolysis, thereby allowing tumor cells to thrive [35, 36]. Hypoxia-triggered exosomes transmit cisplatin resistance to sensitive NSCLC cells through delivery of PKM2 [22]. PKM2 facilitates HSP90-independent stability of IGF-1R precursor protein as well as enhances growth of cancer cells in a hypoxic environment [36]. eEF2 kinase-triggered inactivated STAT3 may inhibit the proliferative capacity of NSCLC cells through phosphorylation of PKM2 [37]. TEAD4 enables to enhance NSCLC progression with PKM2-mediated glycolysis [38]. Silencing PKM2 may retrain tumor growth as well as invasion in NSCLC [39]. Altogether, accumulated pieces of evidence have provided the basic data required for PKM2-targeted gene therapy. Sal B may lower PKM2 expression and its phosphorylation in NCI-H2030 and NCI-H1650 cells.

LDHA and GLUT1 are crucial components of the glycolytic signaling [40]. LDHA [41] and GLUT1 [42] both display upregulation in NSCLC than normal tissues, which enable to independently predict undesirable clinical outcomes. LDHA activation may enhance the growth of NSCLC [43], and miR-449a weakens LDHA-independent glycolysis to strengthen the sensitivity of NSCLC cells to ionizing radiotherapy [44]. NSCLC growth and radiotherapy resistance depend upon GLUT1-independent glucose uptake in tumor-associated neutrophils [45]. LDHA and GLUT1 expression was decreased by Sal B in NCI-H2030 and NCI-H1650 cells, demonstrating the inhibitory function of Sal B in glycolytic signaling of NSCLC cells. Metabolic reprogramming represents a hallmark of cancer metastases [46, 47], and tumor cells manipulate the metabolic profiles to meet the dynamic energy demands of the tumor microenvironment [48–50]. The data showed that Sal B enabled to weaken NSCLC metastasis with PKM2-independent metabolic reprogramming. In future studies, in vivo experiments are required for demonstrating the therapeutic efficacy of Sal B in NSCLC metastasis.

## 5. Conclusion

Altogether, this study provided evidence that Sal B was capable of weakening NSCLC metastasis via PKM2-independent metabolic reprogramming, shedding light on the promising therapeutic usage of Sal B in NSCLC therapy.

## Data Availability

The datasets analyzed during the current study are available from the corresponding author on reasonable request.

## Abbreviations

NSCLC: Non-small-cell lung cancer
PKM2: Pyruvate kinase M2
Sal-B: Salvianolic acid B
FBS: Fetal bovine serum
CCK-8: Cell counting kit-8
p-PKM2: Phosphorylated PKM2
LDHA: Lactate dehydrogenase A
GLUT1: Glucose transporter 1
OCR: Oxygen consumption rate
ECAR: Extracellular acidification rate
FCCP: Carbonyl-cyanide p-trifluoromethoxy phenylhydrazone
antiA: Antimycin A
Rot: Rotenone
2-DG: 2-Deoxyglucose
ANOVA: One-way analysis of variance.
